# What Makes an Effective Chief of Pediatric Cardiology: Insights from Chiefs of Programs Globally

**DOI:** 10.1007/s00246-025-04002-4

**Published:** 2025-08-22

**Authors:** Michael E. Kim, Lars Idorn, Sandra S. Mattos, Worakan Promphan, Anne Dubin, Michael Cheung, Francesca Raimondi, John Lawrenson, Damien Kenny, Babar Hasan, Shubhayan Sanatani, Damien Bonnet, Tom Gentles, Aaron Bell, Shubhika Srivastava, R. Krishna Kumar, Joseph Rossano, Colin J. McMahon

**Affiliations:** 1 Department of Critical Care Medicine, The Hospital for Sick Children, Toronto, Canada; 2 Department of Pediatric Cardiology, Rigshospitalet, Copenhagen, Denmark; 3 Maternal-Fetal and Pediatric Cardiac Unit, Royal Portuguese Hospital, Recife, Brazil; 4 Department of Pediatric Cardiology, Queen Sirikit National Institute of Child Health, Bangkok, Thailand; 5 Department of Pediatric Cardiology, Lucile Packard Children’s Hospital, Stanford, CA USA; 6 Department of Pediatric Cardiology, Royal Children’s Hospital, Melbourne, Australia; 7 John Paul II Children’s Hospital, Bergamo, Italy; 8 Paediatric Cardiology Service of the Western Cape, Red Cross War Memorial Children’s Hospital and Tygerberg Hospital, Cape Town, South Africa; 9 Department Pediatric Cardiology, Children’s Health Ireland at Crumlin, Dublin 12, Ireland; 10 Department of Pediatric Cardiology, Aga Khan University Hospital, Karachi, Pakistan; 11 Division of Cardiology, Department of Pediatrics, Children’s Heart Center, British Columbia’s Children’s Hospital, Vancouver, BC Canada; 12 Department of Pediatric Cardiology, Necker Enfants, MC-3, Paris, France; 13 Department of Pediatric Cardiology, Starship, Auckland, New Zealand; 14 Evelina Children’s Hospital, London, England, UK; 15 Nemours Children’s Heart Center, Nemours Children’s Health and Thomas Jefferson University, Wilmington, Delaware USA; 16 Amrita Institute of Medical Sciences and Research Center, Cochin, Kerala India; 17 Department of Pediatric Cardiology, Children’s Hospital of Philadelphia, Philadelphia, PA USA; 18 University College Dublin School of Medicine, Belfield, Dublin 4, Ireland

**Keywords:** Congenital Heart Disease, Chief, Leadership, Pediatric cardiology program, Collaboration

## Abstract

While the position of a pediatric cardiology chief is seen as the apex of one’s academic career, its roles, responsibilities, and qualifications are not well defined in literature. We previously surveyed North American pediatric cardiology chiefs to characterize these qualities. It is not known if these findings apply across programs around the world. This study was done to gain further insight of the role and attributes of a pediatric cardiology chief beyond North American programs by surveying those who are currently serving the position around the world. This was a mixed-methods study that sent a survey out to pediatric cardiology chiefs from around the world. The survey was a semi-structured questionnaire independently reviewed by three cardiologists. The survey included items rated on a 5-point Likert scale, open-ended prompts, and targeted questions regarding the chief’s role and qualifications. Thirty two pediatric cardiology chiefs from 23 countries completed the survey. The four most important traits for the role of chief included communication, honesty, work ethic, and management skills. During the chief’s tenure, 60–75% of responses noted a change in faculty and trainee work ethic and 50–60% of responses also reported increased expectations from patient families and hospital administration. Thematic analysis on the challenges and success of running a department revealed the importance of building a well-functioning team and individuals possessing clinical excellence and a strong identity. This global survey captures current perspectives on the role of a pediatric cardiology chief across multiple healthcare systems and evolving practices. Awareness of such key attributes may help promote effective leadership skills as chiefs of pediatric cardiology.

**Supplementary Information:**

The online version contains supplementary material available at 10.1007/s00246-025-04002-4.

## Introduction

The departmental chief position is often the pinnacle of one’s academic career in medicine [1–4]. Little is documented in the literature with regard to the role responsibilities, skill requirements, and characterization of who is fit best to lead. The role is a pivotal in establishing departmental culture, developing faculty, and ensuring provision of excellent patient care [5, 6]. We previously examined attributes and challenges North American pediatric cardiology chiefs reported and the evolution of the role working with varied healthcare models, program expansion, revenue targets, patient outcome excellence, and collaboration with subspecialties and corporate suite models [7–11].

We wanted to expand our query to determine if these qualities resonated with other pediatric cardiology leaders on a global scale. We sought to assess how differing healthcare systems, governments, and cultures could potentially influence the position [12, 13]. The objectives of this study were to identify key attributes, challenges, and perceptions of pediatric cardiology chiefs on a global scale and to compare the data with a prior study with exclusively North American programs [1].

## Methods

This was a mixed-methods study with grounded theory employing both quantitative and qualitative components using a semi-structured questionnaire which was approved by three independent pediatric cardiologists with professional degrees in medical education. This was utilized in a prior study focused on North American pediatric cardiology chiefs [1]. This survey was created in SurveyMonkey to aid ease of completion by respondents. Invitations to participate in the survey were distributed via email to pediatric cardiology chiefs identified across North America, South America, Europe, Africa, South Asia, and Australia/New Zealand.

Programs and individuals were invited semi-randomly to achieve proportional representation of pediatric cardiology centers globally. Need for consent to perform this study was waived by the Ethics department at Children’s Health Ireland (Crumlin, Dublin, Ireland). Consent was requested from all respondents to complete the survey and to facilitate the results of the study being disseminated in a peer-reviewed pediatric cardiology journal. Surveys provided the option for respondents to be identified if they wished to be acknowledged. Items within the survey were not mandatory, and participants were given the option to opt out of responding certain questions. All survey data were stored securely in a password protected document accessible only by the study team.

### Statistical Analysis

Demographics and descriptive statistics including Likert scale responses from the survey were summarized by medians with interquartile ranges and frequencies with percentages. Within the survey, participants ranked items which were then calculated by means with standard deviations. The open-ended prompts were manually coded by two independent reviewers which then were compiled into themes. Thematic analysis was done by open coding and utilizing inter-rater agreement with disputes resolved via a systematic approach with open discussion, reflection, and careful review/refinement of code definitions.

## Results

### Demographics

**Fig. 1:**
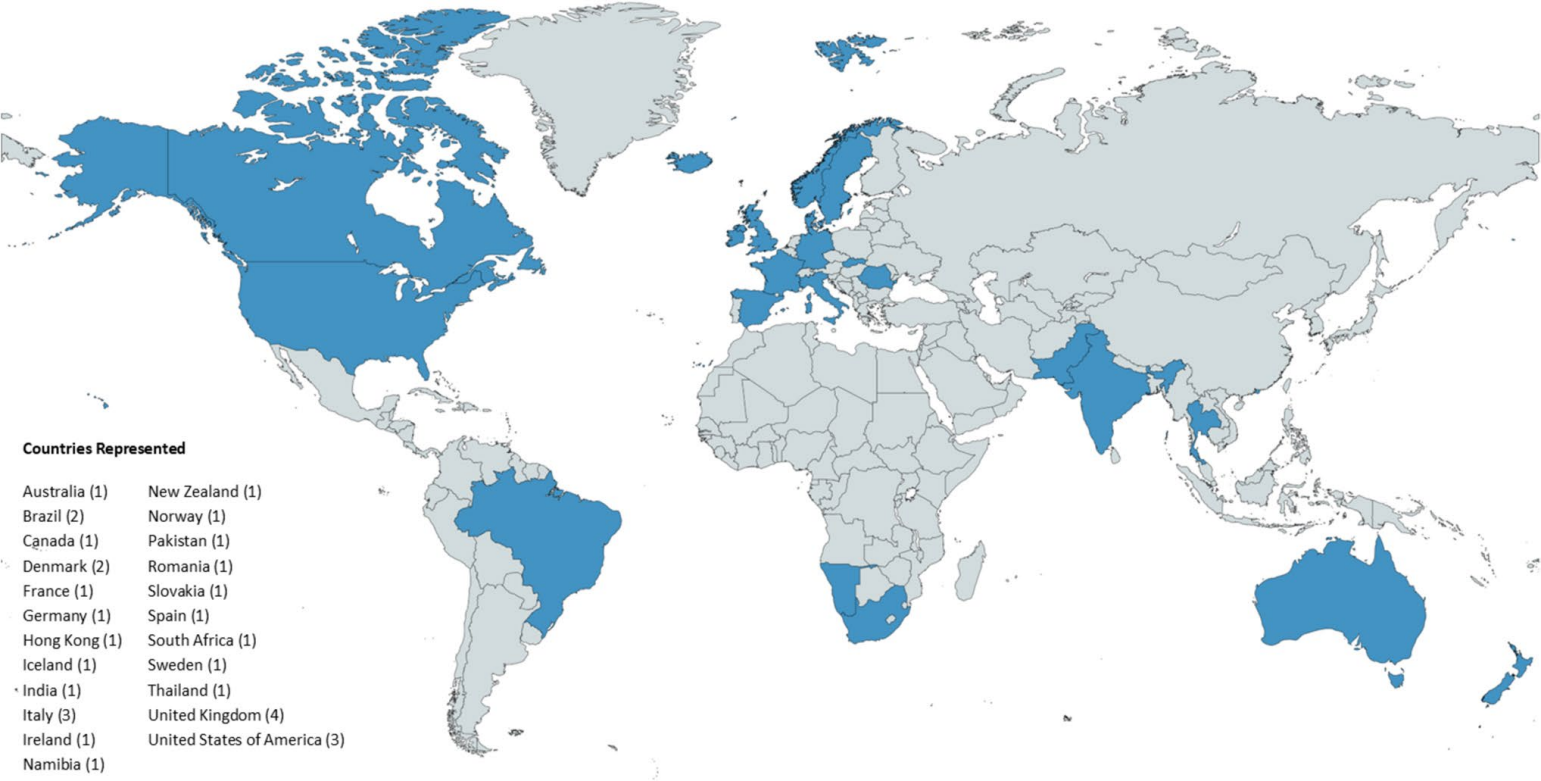
Global map depicting participant representation by country

The survey was distributed to chiefs in pediatric cardiology centers around the world which cis visualized in Fig. 1. Demographics included the number of years in practice as a pediatric cardiologist, number of years serving as pediatric cardiology chief, and whether this was their first chief position (Table 1). In total, 32 chiefs out of 49 who were invited (65% response rate), representing 23 countries, responded. Approximately, 22% (7) of respondents were female.

**Table 1:** Participant demographics

Item	Participants %, (*n* = 32)
Years in practice, median (IQR)	23 (17, 30)
Years served as chief, median (IQR)	5 (3, 12)
First time position, % (*n*)	91 (29)
Cardiopulmonary bypass cases a year	
101–250	34 (11)
251–400	41 (13)
401–600	9 (3)
601–800	9 (3)
801–1000	3 (1)
Department faculty size, median (IQR)	10 (6, 15)

### Key Attributes of Effective Chiefs

The survey tasked participants to rank a list of traits in order of importance for the chief position (Table 2). In summary, the top 4 traits were similar in ranking to the North American pediatric cardiology chiefs in prioritizing communication skills, honesty, hard-working, and management skills. The global survey data then diverged in rankings beyond the top 5 traits aside from “Teacher” and “Reputation” being ranked last in both groups.

**Table 2:** Average ranking of traits important for pediatric cardiology chief role (1 = most important, 14 = least important)

Item	Average rank (SD)
Communication skills	2.9 (2.2)
Honesty	3.2 (2.8)
Hard-working	5.5 (3.1)
Management skills	6 (2.9)
Equitable treatment	6.5 (3)
Effective negotiator	8.6 (3.4)
Decisive	9 (3.8)
Academic excellence	9 (4)
Promoting psychological safety	9.2 (3.7)
Self-awareness	9.2 (4.1)
Conflict resolution	9.4 (3.4)
Humility	9.5 (4.4)
Visionary	9.5 (4.4)
Teacher	10.8 (3.3)
Reputation	11.5 (3.7)

**Fig. 2:**
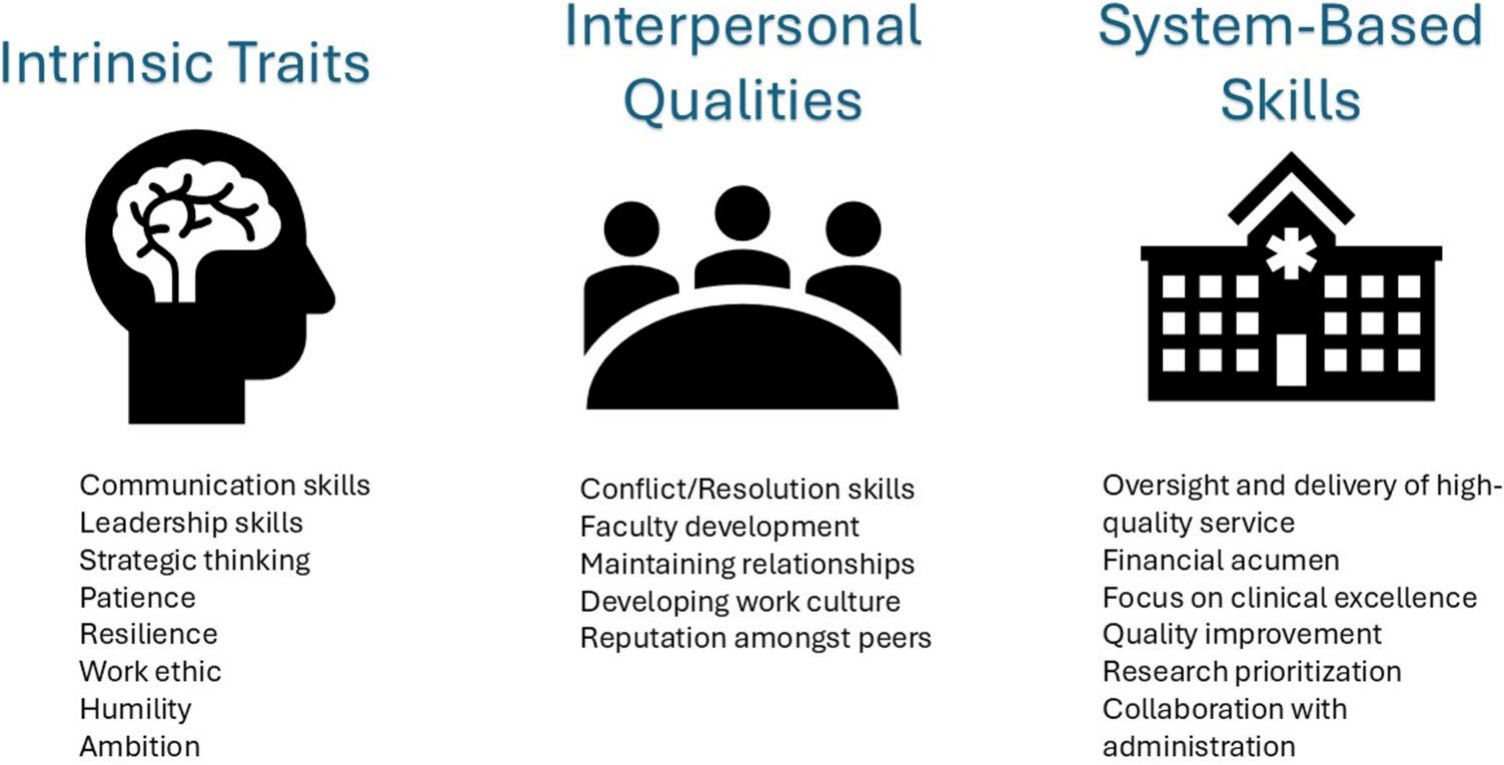
Schema of important attributes and duties of a pediatric cardiology chief

Combining the attribute ranking with additional open-ended responses led to the creation of three distinct categories based on the traits and responsibilities of the chief position (Fig. 2). Specifically, the lists were also organized in descending order of the most referenced items within each category. The three categories—intrinsic traits, interpersonal qualities, and system-based skills—all emphasized attributes pertaining to a high-performance team (communication skills, conflict/resolution skills, oversight/delivery of high-quality service).

The majority of participants agreed for a term limit with the pediatric cardiology chief role, although there was also a quarter of participants who favored the possibility of longer terms should the group favor the current leader (Table 3). The overwhelming majority of respondents favored the concept of succession planning. When asked if individuals started their career over if they would serve as chief again, there was near unanimous agreement. When asked if formal education in business management or administration would be helpful there was an even split among respondents.

**Table 3:** Responses pertaining to chief position longevity and succession

Item	Response, % (*n*)		
	Yes	Depends	No
Should the chief position rotate every 5–10 years? (*n* = 29)	44 (14)	28 (9)	22 (7)
Do you believe succession planning is important as a part of the chief role? (*n* = 30)	90 (28)	–	10 (3)
If you started your career over, would you serve as chief again? (*n* = 31)	94 (30)	3 (1)	3 (1)
Do you think formal management education (i.e., MBA) is important to be effective in the role? (*n* = 29)	40 (12)	–	60 (18)

### Likert Scale Responses

**Fig. 3:**
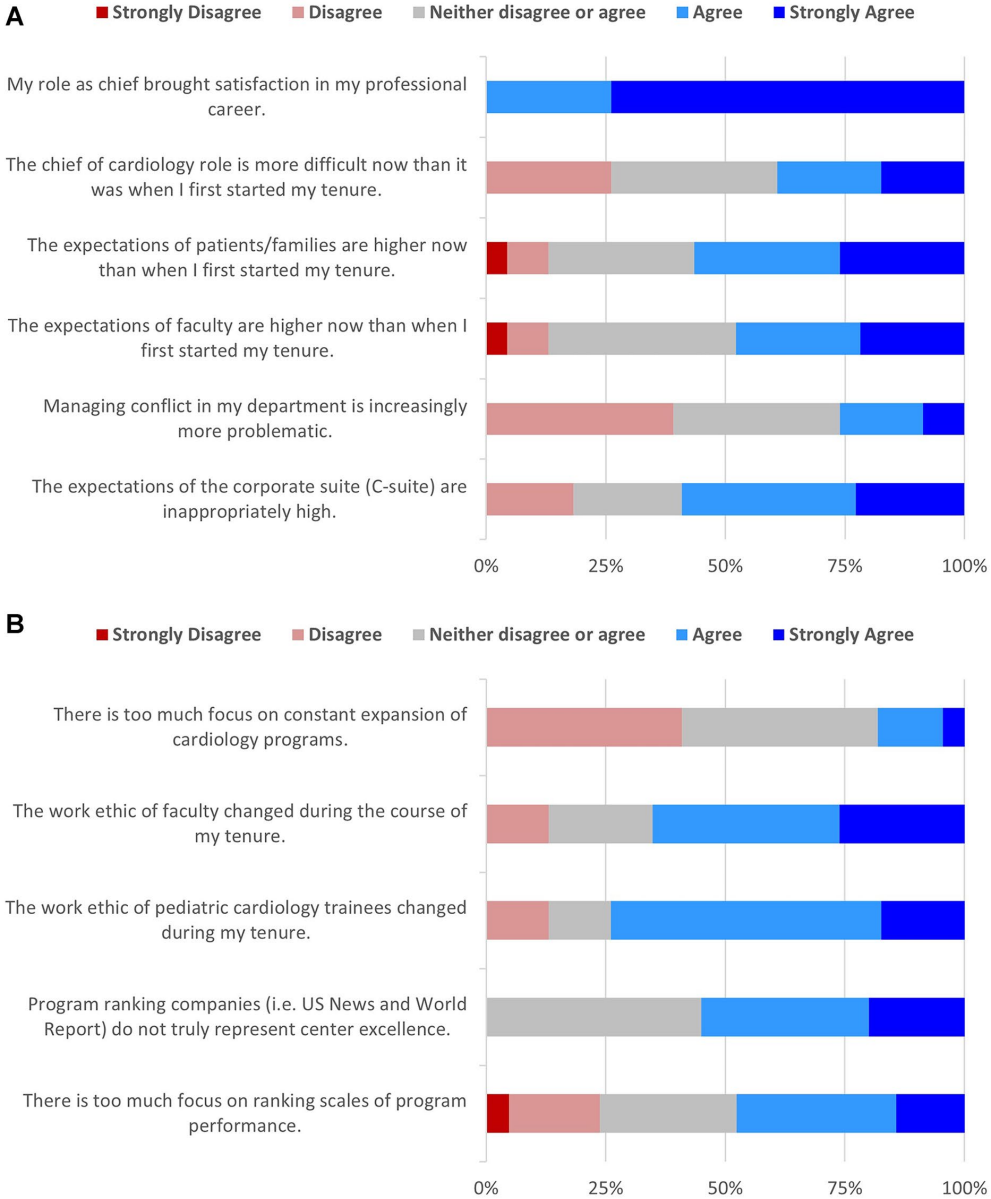
**A** and **B** 5-point Likert scale survey responses on chief position and perceptions

Survey items were rated on a 5-point scale which is summarized in Fig. 3A and B. Respondents reported satisfaction in their tenure as chief. Compared to data from North American chiefs [1], participants did not appreciate higher expectations from faculty and managing the department, although they recognized higher pressure from patients/families and the corporate suite. Like the North American counterparts, there is a similar commentary of changes in work ethic among faculty and trainees which was cited as mainly due to more emphasis on work-life balance with several expounding that this was not a bad thing. Finally, when tasked in responding about items pertaining to program expansion and program ranking, global respondents did not resonate with these items compared to North American counterparts and faced different challenges (i.e., staffing issues, different healthcare system models).

### Qualitative Prompts and Themes

We included open-ended prompts to further delineate the priorities, challenges, and benefits of serving in the role of a pediatric cardiology chief in a global context. Two main themes emerged from the coded data which included (1) Building and developing a well-functioning team is essential for long-term success of the program; (2) The role requires a strong sense of self and responsibility to others as well as oneself in terms of knowing one’s limitations and strengths.

### Theme 1: Teamwork, Building a Team, and Team Management

The first theme overwhelmingly (over 75% of responses) was repeated by participants in terms of stressing the importance of teamwork, building a well-functioning team, and the challenges of managing a dysfunctional team which all contribute to the outcomes of a program. These include the responsibilities of the chief recruiting and hiring the right people as well as being responsible for developing faculty and the culture of a program well. It is also important that a recurring item with this theme also included the need to bring a team together in a single vision/mission.

...To establish a [program] where colleagues feel welcome and are devoted to aim for the highest clinical and scientific standards within pediatric cardiology…

...Choose your best team. Realize who has the potential and how to achieve the best results as a team. Make everybody shine—see the potential in your employees, give them time and room to evolve…

...Keeping a big team happy and together is difficult! Be visible and approachable. Be kind…

...Building a team is more important than excellence. A mixed team of both sexes is important. Different personalities are needed in different areas of cardiology…

...Developing and sustaining a harmonious team is the most critical aspect of running a pediatric heart program…all the members of the team should deeply resonate with the mission and vision of the program that needs to be articulated with great clarity…

### Theme 2: Introspection and Identity

The second theme resonated with much more introspective insights from respondents who emphasized elements that were not readily visible to their team members or patients. Over half of the responses contained phrasing or sentiments on the importance of leaders needing to have a strong sense of self and ability to reflect. It was apparent that this need to self-actualize was a crucial step in succeeding in the role.

...Be loyal to yourself and your team. Fight for what you think is the best for your team and the patients—even if [others] want you to do differently. Do not be afraid or hesitate to take an unpleasant decision if you know or think it is the right thing to do…

…Leadership can only be sustained if the leader leads by example…

While not as high of a frequency in responses to warrant a third theme, there were many comments surrounding the clinical competency and skills as a physician that respondents felt were important in being a successful chief:

...The essential attributes would include high degree of clinical competence, excellent communication and teaching skills, a deep understanding of what it takes to be a good mentor, an excellent grasp on the healthcare landscape of the region…it is also important to develop excellent working relationships with other providers…

...be a very good doctor in pediatric cardiology, probably the best...be a good leader…be capable of coordinating a team… be an example for others…

...I think the perfect chief needs professional insight broadly in pediatric cardiology as well as good leadership abilities…

## Discussion

Despite the wide range of program sizes, models, and healthcare systems compared to the North American model [1], there were a lot of similarities when assessing key attributes for the pediatric cardiology chief role with the main crux revolving around communication and interpersonal skills (Table 2). There was a higher emphasis from the global counterparts on a leadership model emulating more similarly to a “role model” as opposed to a “servant leader” which was more common in North American programs, emphasizing the need for clinical expertise in the field. Differentiating the two is important—the role model paradigm focuses on leaders showcasing desired behaviors/values for individuals to follow as opposed to a servant leader who prioritizes the needs and development of team members actively by creating a supportive environment to achieve those goals.

There was an emphasis on the challenges and pressures of the chief position for program excellence which stemmed from the ability of a team to function at a high level. This is dissimilar from the North American counterparts where they felt the push for program excellence was propagated by the need for program expansion. It is reassuring that ultimately respondents were very satisfied with the role regardless of nationality or program structure which again emphasizes that the role possesses more positive outcomes than detriments.

In terms of succession planning, while there was near unanimous sentiment in its importance, there was less clarity in the need for term limits. Participants discussed the need for flexibility if the group agreed to allow the chief to continue serving the position whether it is due to excellence in leadership or circumstantial.

When examining how to best construct the “perfect” pediatric cardiology chief based on the responses, there is an emphasis on the chief’s ability to build and develop a team, possessing strong interpersonal skills and demonstrating clinical excellence. Interestingly when considering the importance of formal training in business administration the majority of participants felt it was not warranted—however, we also did not examine how many of the participants already received formal training and whether this affected their response.

Similar to prior studies, there is a continued lack of representation in leadership with respect to diversity, equity, and inclusion—in particular gender equity [1, 14–22]. While those qualified for role often take the position, there are conscious and unconscious biases influenced by culture (workplace and societal) and interpersonal dynamics that should be acknowledged in the process so that influential leadership positions are representative of the population [23].

Since the Covid-19 pandemic, international collaboration and communication has risen dramatically which has opened several new potential opportunities within pediatric cardiology. For a highly subspecialized field, shared information and practices can continue to advance the field and create dialogue which often may occur at the direction of the chief. Proper leadership education, training, and intentionality are important to build functioning partnerships globally [24].

We therefore have proposed several components for ongoing efforts on a global scale for pediatric cardiology programs and chiefs to address our findings:

*Proposed Support for Chiefs of Pediatric Cardiology Programs:*

1. Greater research on the role of the chief position globally including leadership training, education, and collaborative efforts
2. Increased support for women in leadership roles and programmatic sensitivity toward gender equity
3. Development of formal workshops to facilitate forums in which chiefs can share challenges with the role
4. Establishment of regular online meetings for chiefs to discuss challenges and successes
5. Innovation in pediatric cardiology fellowship education with tracks for those interested in administration and leadership

## Limitations

There are several limitations that should be acknowledged in this study. This questionnaire, while reviewed by multiple cardiologists, is not a widely validated instrument as one does not currently exist [1]. We expanded the voice of the chiefs in this study by expanding the scope of programs on a global scale—we did not completely capture every country nor did we achieve a high number of respondents, although we attempted to control the representation from certain regions due to the number of programs. We also did not include topics surrounding financial compensation and business models which are influential factors in the role and can vary significantly across different health systems—this could be addressed in future studies. While the perspective of the chiefs is highly valued in this research question, other stakeholders within the department are likely just as important (i.e., faculty).

## Conclusion

There is significant variability in the pediatric cardiology chief position on a global scale. There were enough differences highlighted to challenge the notion that while North American programs academically represent an archetype for clinical excellence, there is much to be gained and appreciated from a global perspective. Future studies can examine these similarities and differences at a deeper level whether it be a direct comparison of healthcare models, program structure, and center volume.

## Supplementary Information

Below is the link to the electronic supplementary material.

Supplementary file1 (DOCX 18 KB)

## Data Availability

No datasets were generated or analysed during the current study.
