# Supplementary material for: What Makes an Effective Chief of Pediatric Cardiology: Insights from Chiefs of Programs Globally
Source: Pediatr Cardiol. 2025 Aug 22;47(5):2104–11. doi: 10.1007/s00246-025-04002-4 (PMC13144182; doi:10.1007/s00246-025-04002-4)
Supplement: Supplementary file 1 — Supplementary file1 (DOCX 18 KB) [file 246_2025_4002_MOESM1_ESM.docx]

Global Chief of Pediatric Cardiology Survey Instrument

1. By filling out this survey I agree with the information that is collected and its findings to be presented and/or published in a peer review journal.
2. What is the name of your centre of practice?
3. Where is your country of practice?
4. How many paediatric cardiologists work in your centre?
5. How many cardiopulmonary bypass procedures are performed per year at your centre?
6. How many years in paediatric cardiology practice?
7. How many years as chief of paediatric cardiology service?
8. Is this your first post as a chief of paediatric cardiology?
9. What are the important roles a chief in paediatric cardiology needs to fulfil?
10. What are important characteristics for a person to succeed as chief in paediatric cardiology?
11. Rank these characteristics in order of importance starting with the highest being 1 and the lowest 15:

Communication skills
Honesty
Hard-working
Equitable treatment
Academic excellence
Management skills
Reputation
Teacher
Effective negotiator
Promotes psychological safety
Self-aware
Decisive
Able to resolve conflict
Visionary
Humility

1. What are three important lessons you learned during your term as chief of paediatric cardiology?
2. Should the role of chief be for life or rotate every 5-7 years?
3. Is there an optimal age to be appointed as chief of pediatric cardiology?
4. What challenges did you face as a chief of paediatric cardiology?
5. What benefits did you experience as chief of pediatric cardiology?
6. Do you believe succession planning to be an important part of the chief role?
7. Do you think formal education in management (MBA or Masters in Management) is important to be effective in the role?
8. How does leadership factor into being an effective chief of pediatric cardiology?
9. What specific leadership skills do you think are/were important in your tenure as chief?
10. Would you undertake the role of chief of pediatric cardiology if starting your career again?

**5-point Likert Scale Items (Strongly Disagree to Strongly Agree).**

1. My role as chief brought satisfaction in my professional career.
2. The chief of cardiology role is more difficult now than it was when I first started my tenure.
3. The expectations of patients/families are higher now than when I first started my tenure.
4. The expectations of faculty on you are higher now than when I first started my tenure.
5. Managing conflict in my department is increasingly more problematic.
6. The expectations of the corporate suite (C-suite) are inappropriately high.
7. There is too much focus on constant expansion of cardiology programs.
8. The work ethic of faculty changed during the course of my tenure.
9. The work ethic of pediatric cardiology trainees changed during my tenure.
10. Program ranking companies (i.e. US News and Report) do not truly represent centre excellence.
11. There is too much focus on ranking scales of program performance.
12. What would you like to see as your legacy after completing the chief position?
13. What important points of discussion relevant to your role do you feel are important?
14. Did reflecting through this survey help in any capacity?
15. Any important points you think we missed?
16. Please feel free to include your name if you wish to be acknowledged in the paper.
